# EEG-based trial-by-trial texture classification during active touch

**DOI:** 10.1038/s41598-020-77439-7

**Published:** 2020-11-27

**Authors:** Safaa Eldeeb, Douglas Weber, Jordyn Ting, Andac Demir, Deniz Erdogmus, Murat Akcakaya

**Affiliations:** 1 Electrical and Computer Engineering Department, Swanson School of Engineering, University of Pittsburgh, Pittsburgh, PA USA; 2 Bioengineering Department, Swanson School of Engineering, University of Pittsburgh, Pittsburgh, PA USA; 3 Electrical and Computer Engineering Department, Northeastern University, Boston, MA USA

**Keywords:** Biomedical engineering, Neuroscience

## Abstract

Trial-by-trial texture classification analysis and identifying salient texture related EEG features during active touch that are minimally influenced by movement type and frequency conditions are the main contributions of this work. A total of twelve healthy subjects were recruited. Each subject was instructed to use the fingertip of their dominant hand’s index finger to rub or tap three textured surfaces (smooth flat, medium rough, and rough) with three levels of movement frequency (approximately 2, 1 and 0.5 Hz). EEG and force data were collected synchronously during each touch condition. A systematic feature selection process was performed to select temporal and spectral EEG features that contribute to texture classification but have low contribution towards movement type and frequency classification. A tenfold cross validation was used to train two 3-class (each for texture and movement frequency classification) and a 2-class (movement type) Support Vector Machine classifiers. Our results showed that the total power in the mu (8–15 Hz) and beta (16–30 Hz) frequency bands showed high accuracy in discriminating among textures with different levels of roughness (average accuracy > 84%) but lower contribution towards movement type (average accuracy < 65%) and frequency (average accuracy < 58%) classification.

## Introduction

Haptics technology allows one to perceive tactile information through their sensation. Researchers developed haptic devices that are capable of generating touch sensations^1–4^. Teleoperation, neuroprothestics, surgical training of physicians in virtual environment and remote control of robotic arms are different applications of haptics^5–9^.

Sense of touch, through the somatosensory system, allows us to identify, grasp, evaluate, and manipulate objects^10^. Sensory information is retrieved through a complex network of nerve endings, sensory neurons and touch receptors in the skin. This information is then transported to the primary (S1) and secondary (S2) somatosensory cortex^11^. The somatosensory system involves two main neural inputs, cutaneous and kinesthetic, which contribute to the touch sensation process. Mechanoreceptors in the epidermis and dermis layers of the skin are the main contributors in sensations of touch. The mechanoreceptors of the index fingertip are of four types with small and large receptive fields and have slow and rapidly adapting receptive fields which enable high spatial resolution^11,12^.

Over the past years, the terms passive and active touch have been given a variety of definitions. In this paper, we used the definition introduced by Lederman^13^, where active touch of tactile stimuli is a dynamic process, which typically involves active movement against a surface to explore its texture. It was shown that active movement enhances the sensory inputs and provides rich information about the surface properties, while, without this movement, it is very difficult to discriminate between similar textures^10,14,15^. During active stimulation of the skin, both slowly and rapidly adapting mechanoreceptors contribute to the touch sensation process. Mechanoreceptors in the skin send signals to the thalamus which projects to the primary somatosensory cortex^16,17^. The somatosensory system is organized in a contralateral fashion, where the signals sent from the right side of the body project to the left hemisphere and signals from the left side project to the right hemisphere. Blatow et al.^17^, performed a tactile stimulation study on the fingers of healthy subjects during tactile stimulation of both right and left fingers. They showed higher activation in the contralateral side of the somatosensory cortex during stimulation.

Until recently, most studies attempting to investigate the cortical activity associated with touching textured surfaces while recording EEG have involved passive touch only^18–20^. Passive touch indicates that the participant’s index finger is at rest during vibro-tactile stimulation. A device that provides a dynamic passive stimulation that mimics the movement of sliding an object against a participant’s finger while recording EEG was introduced^18^. This study showed an increase in the power of the theta band (4–7 Hz) for 500 ms after the stimulus onset, followed by a decrease in the power of the alpha band. A later study by the same group showed a linear decrease in the alpha band amplitude with increasing roughness of the stimulus^20^. A significant relation between the power in the beta band and the discrimination between pleasant (soft) and rough textures have been shown in response to different natural textures^19^. A recent study found greater activation for active touch while studying the underlying neural mechanisms of active and passive touch^21^.

Both active and passive touch activated similar cortical areas in the brain. An attempt to compare the cortical activity involved in active and passive touch using EEG was proposed by Monungou et al.^22^. They used a device that produces ultrasonic waves which modulate friction at a frequency of 11 Hz, producing a tactile percept that mimics a square-wave grating, during both active and passive touch. A force feedback-controlled robot was used to reproduce, in the passive touch condition, the exact movements and normal force produced during the active touch condition. The somatosensory Evoked Potentials (SS-EPs) were recorded, and the brain activities at the frequency of the friction modulation in both active and passive touch conditions were compared^22^. Analysis of recorded force data confirmed that both passive and active touch conditions were matched. The recorded EEG over the central and parietal electrodes, contralateral to the stimulated fingertip, showed that the measured SS-EPs were highly similar in both conditions. In summary, recent studies of the EEG response to tactile stimuli of either natural or synthetic textured surfaces involve passive touch only^18,23^, except for one attempt by Monungou et al.^22^. None of the studies have combined active touch with single trial EEG-based texture classification^22^. Additionally, to the best of our knowledge, there is no study of the influence of movement type and frequency level on the EEG response during active touch exploration.

The main research objective of this study is to identify and analyze salient spectral and temporal EEG features that could differentiate among textured surfaces with different levels of parametrically controlled roughness while minimizing the effect of movement frequency and type conditions on the overall surface identification accuracy. These features were chosen to classify textures based on a single trial and not on the average EEG response over all trials per condition. The selected set of features will then be used to develop a closed loop feedback system. This system will mimic the sensation of various textured surfaces during different applications such as: surgical training of physicians in virtual environment, remote control of robotic arms and teleoperations.

## Results

### Texture classification

**Figure 1:**
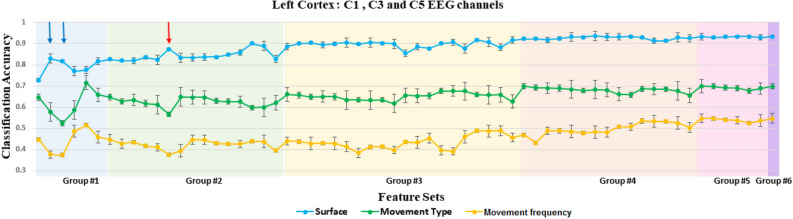
The mean and standard deviation of the classification accuracy of each classification problem (texture, movement type and frequency) calculated at each feature set and averaged across all the participants and all conditions and averaged over the three EEG channels (C1, C3 and C5). Each group of feature sets is highlighted by different background color. The selected set of features are marked by the blue and red arrows.

The results of the systematic feature selection, which is explained in details under the classification subsection of the “Materials and methods” section, showed that the spectral EEG features, (the total power in the mu (8–15 Hz) and beta (16–30 Hz) frequency bands), showed high accuracy in discriminating among textures with different levels of roughness while movement type and frequency classification accuracies were low, see Fig. 1. As also explained in “Materials and methods” section, six different features were used for this analysis, (the total power in the theta band, the total power in the mu band, the total power in the beta band, the total power in the gamma band, the average EEG amplitude and the P300 response). Figure 1 demonstrates the changes in the accuracy of texture classification, movement type classification, and movement frequency classification as a function of different subsets of the considered six features. Each group of features is highlighted by different background color. Each mark indicates the mean and standard deviation of the accuracy of each classification problem, texture (blue marks), movement condition (green marks) and movement frequency level (orange marks) calculated at each feature subset and averaged across all the participants and all conditions for the three EEG channels (C1, C3 and C5), as shown in Fig. 1. Each group is highlighted by different background color. Since the minimum classification accuracies for movement type and frequency classifications are obtained in groups 1 and 2, we here focus on the results obtained in these two groups. Moreover, even though there are fluctuations in the accuracies obtained for groups 3, 4, 5 and 6, there is almost a plateau type of behavior in these groups.

**Figure 2:**
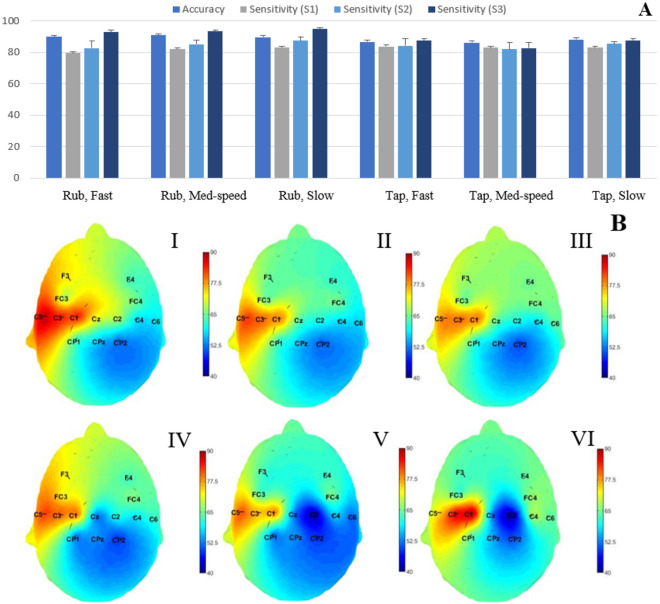
(**A**) The mean and standard deviation of the accuracy, sensitivity of each class (flat, medium-rough and rough surfaces) calculated at the electrode channel with the highest accuracy value and averaged across all participants for each condition. S1: flat, S2: medium-rough and S3 is the rough surface. (**B**) Scalp topography for the average accuracy values at each electrode location across all participants, where each condition is as follows, I: rub at fast movement frequency, II: rub at medium movement frequency, III: rub at slow movement frequency, IV: tap at fast movement frequency, V: tap at medium movement frequency and VI is tap at slow movement frequency.

In group 1, both subsets marked by the blue arrows (total power in mu and beta bands) showed high accuracy in discriminating among textures while providing the minimum contribution towards movement type and frequency classification. In group 2, the combination of the features based on the total power in mu and beta, as marked with a red arrow in Fig. 1, increased the classification accuracy for texture identification while the accuracies for other classifications were still low (not minumum but still lower than groups 3, 4, 5, and 6). Based on the observed classification performances in Fig. 1, the rest of the results are based on two-feature subset of the total power in the mu (8–15 Hz) and beta (16–30 Hz) frequency bands as marked with a red arrow in Fig. 1. The electrode locations, C1, C3 and C5, showed the highest accuracy values in discriminating textured surfaces across all touch conditions. However, the electrode location with the highest texture classification performance varied for each participant. In order to determine the highest performance for each participant, we selected the electrode channel that showed highest accuracy and sensitivity and averaged the results across all participants. The mean and standard deviation of the accuracy and sensitivity of the three textured surfaces for the 3-class texture classification across all participants are shown in Fig. 2. A for each movement type and frequency condition. The x-axis represents the touch condition, and the y-axis shows the performance. These accuracy values were calculated at the channel that has the highest accuracy value across channels per participant. Overall, the accuracy of texture identification for the rub condition is higher than the tap condition. Moreover, the accuracy of identification increased with lower movement frequency of interaction and as the roughness of the surface increased.

Also, the comparison between the average classification accuracy values for all the participants across all channels per each condition are shown in Fig. 2B. Specifically, scalp topographies for the accuracy values at each electrode location for each condition averaged across all participants are shown in Fig. 2B. The topographic maps of the classification accuracy show maximal values over the somatosensory region contralateral to the stimulated finger. Specifically, the electrode locations (C1, C3 and C5) showed the highest accuracy values across all touch conditions, as can be seen in Fig. 2B.

### Movement type classification

**Figure 3:**
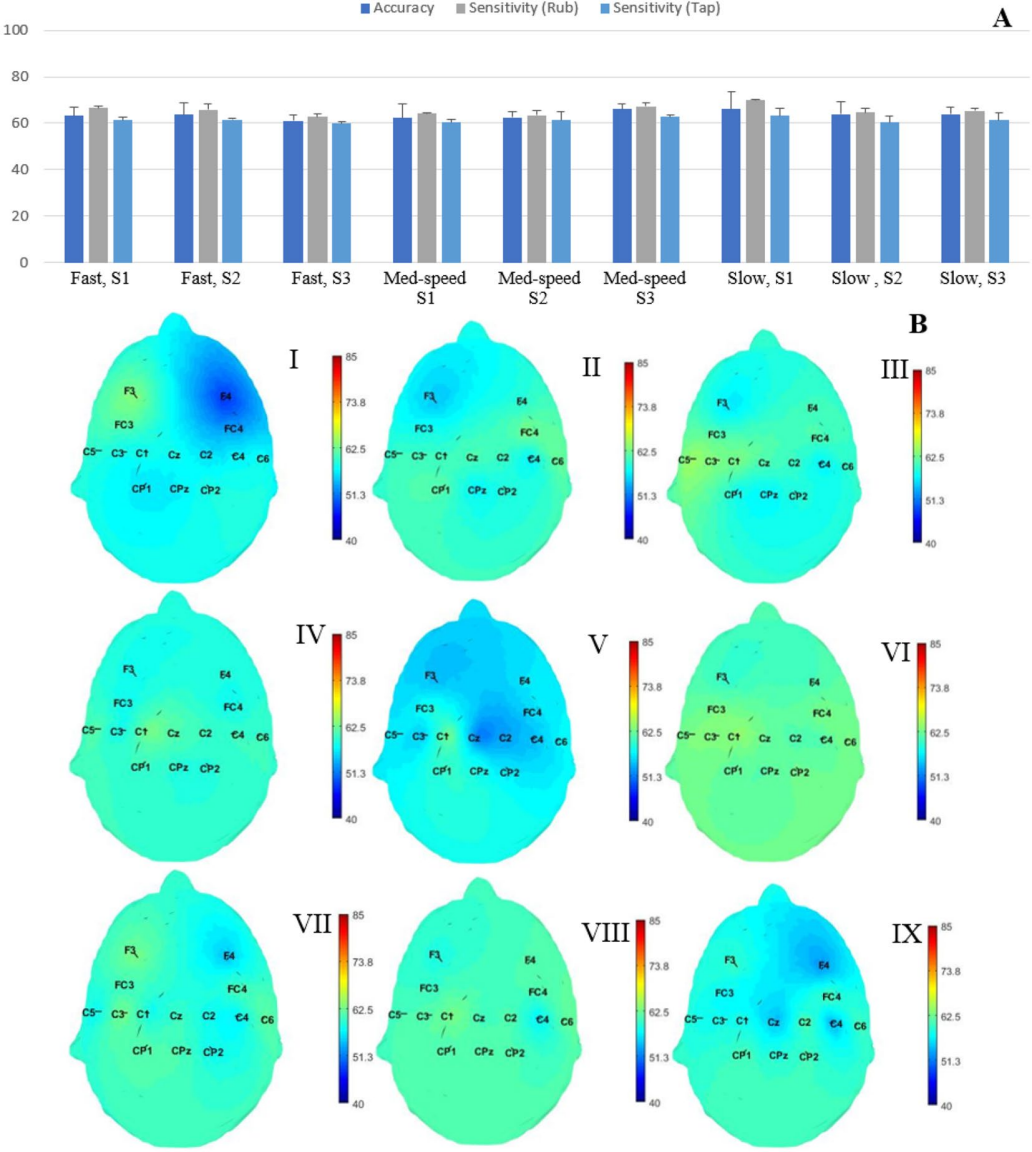
(**A**) The mean and standard deviation of the accuracy, sensitivity of each class (rub and tap) calculated at the electrode channel with the highest accuracy value and averaged across all participants for each condition. S1: flat, S2: medium-rough and S3 is the rough surface. (**B**) Scalp topography for the average accuracy values at each electrode location across all participants, where each condition is as follows, I: flat surface, fast movement frequency, II: med-rough surface, fast movement frequency, III: rough surface, fast movement frequency IV: flat surface at medium movement frequency V: med-rough surface at medium movement frequency , VI: rough surface at medium movement frequency , VII: flat surface at slow movement frequency, VIII: medium-rough surface at slow movement frequency and IX: is rough surface at slow movement frequency.

We aim in this part of the study to minimize the movement type classification while maximizing texture classification, in order to minimize the influence of the movement type on the selected features. Similar to texture classification, the same set of features chosen from the systematic feature selection has been used in classifying movement type, rub vs tap. The results of the movement classification are shown in Fig. 3. The mean and standard deviation of the accuracy and sensitivity of the two movements, rub and tap, for the 2-class classification across all participants are shown in Fig. 3A. The x-axis represents the touch condition and the y-axis shows the performance. Similar to the texture classification problem, these accuracy values were calculated at the electrode location with the highest accuracy value across all channels. The overall average classification accuracy of the movement type classification for all conditions ranges between 60 and 65%. The sensitivity of the rub movement is higher than the tap movements for all the touch conditions (Fig. 3B). The topographic maps showing the comparison between the classification accuracy across all channels per condition are shown in Fig. 3B. The average classification accuracy across all electrode channels are distributed uniformly, with slightly higher values around the frontal and parietal lobes.

### Movement frequency classification

**Figure 4:**
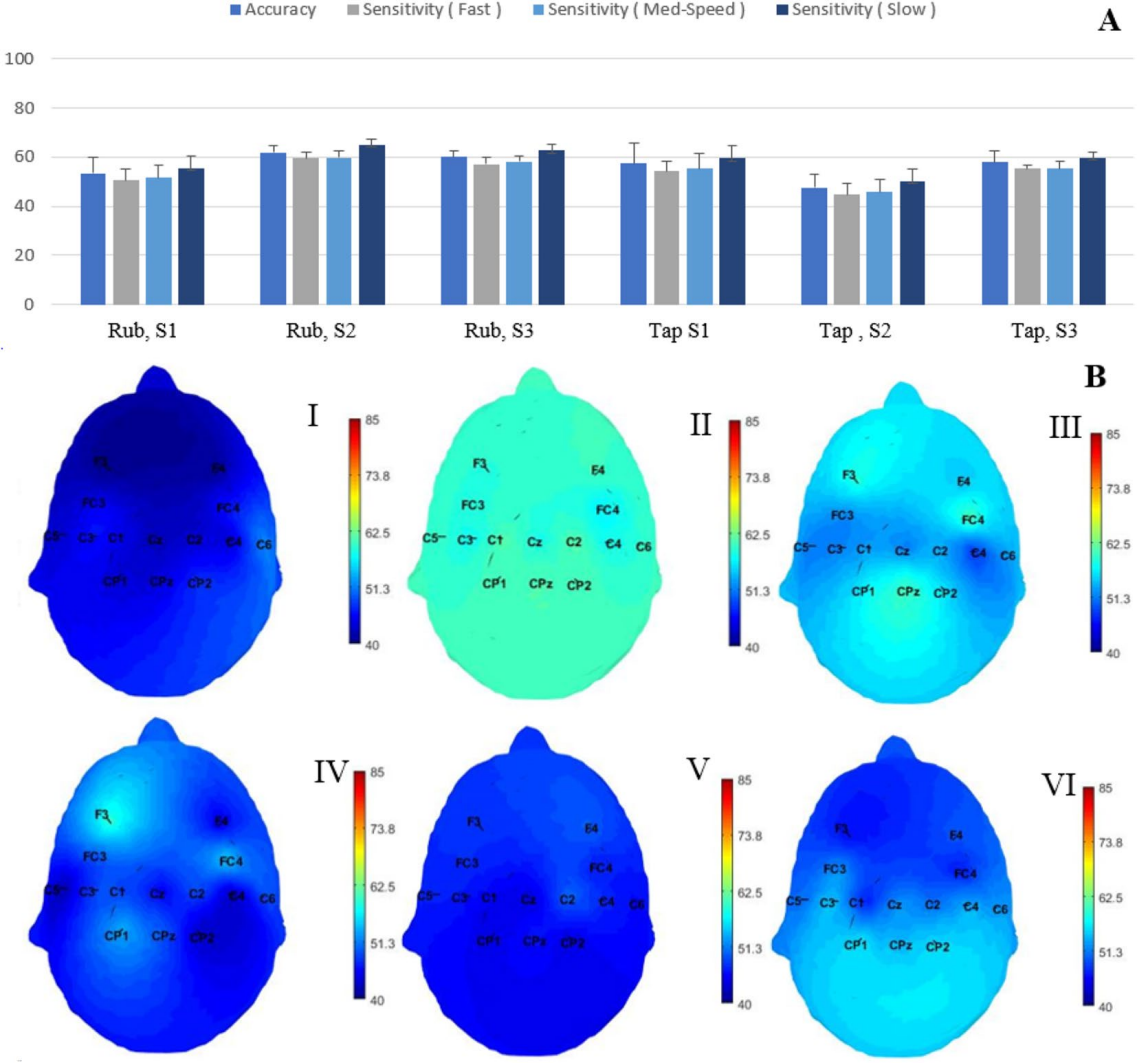
(**A**) The mean and standard deviation of the accuracy, sensitivity of each class (fast, medium movement frequency and slow) calculated at the electrode channel with the highest accuracy value and averaged across all participants for each condition. S1: flat, S2: medium-rough and S3 is the rough surface. (**B**) Scalp topography for the average accuracy values at each electrode location across all participants, where each condition is as follows, I: flat surface, rub, II: med-rough surface, rub, III: rough surface, rub IV: flat surface, tap V: med-rough surface, tap, VI: rough surface, tap.

Similar to movement type classification, our aim is to minimize the movement frequency classification while maximizing the texture classification. The performance of the movement frequency classification is shown in Fig. 4, where the mean and standard deviation of the average accuracy and sensitivity of the three frequency levels across all participants are presented for all the different conditions. Similar to the texture and movement classification problems, these accuracy values were calculated at the highest accuracy value across channels per participant and using the same set of features. Moreover, the topographic maps of the comparison between the classification accuracy across all the channels per each condition are shown in Fig. 4B. The overall average classification accuracy of the movement frequency level ranges between 45 and 58 %. The sensitivity of detecting slow movement frequencies is higher than higher frequency levels (Fig. 4B). The topographic maps demonstrating the comparison between the average movement frequency classification accuracy across all the channels per each condition are shown in Fig. 3B. The average classification accuracy across all the electrode channels are distributed uniformly, with slightly higher values at the following conditions: rubbing medium rough surface, rubbing rough surface and tapping rough surface.

### Texture classification across movement conditions

Additional to the movement type and frequency classification performed, and in order to validate the use of the chosen features (the total power in the mu (8–15 Hz) and beta (16–30 Hz) frequency bands in classifying textures with minimal movement type and frequency influence, three classification problems have been formed. For each movement condition, rub and tap, we combined the data of the three movement frequencies per each class. For example, for the rub movement and for the flat surface, we combined the data observations for the three movement frequency levels forming one class. Similarly, for the medium rough and rough surface, resulting in three class texture classification problem. additionally, we combined all the movement frequency levels and movement types per each textured surface forming three class texture classification problem. The mean and standard deviation of the accuracy, sensitivity of each class, (flat, medium and rough surfaces), averaged across the twelve participants are shown in Table 1. The accuracy of the rub condition is higher than the tap movement condition. The sensitivity of discriminating textured surfaces increases with the increase in roughness level. Moreover, the accuracy of the three classification problems is greater than 80% with low variance. The results of comparing the kernel type used in the SVM classifier for the texture classification across movement conditions can be seen in Table 2.

**Table 1:** The mean and standard deviation of the accuracy, sensitivity of each class (S1: flat, S2: medium and S3: rough surfaces) averaged across the twelve participants.

Condition	Accuracy	Sensitivity	Sensitivity	Sensitivity
		S1 (%)	S2 (%)	S3 (%)
Tap	82.4 ± 0.5	77.85 ± 7.87	83.34 ± 2.45	86.89 ± 3.27
Rub	84 ± 0.1	79.15 ± 4.48	86.21 ± 1.97	88.9 ± 2.84
All movement conditions (type and frequency)	81.3 ± 0.7	75.26 ± 5.38	77.89 ± 1.88	85.98 ± 3.35

**Table 2:** The mean and standard deviation of the accuracy of texture classification across all movement conditions while using Linear, polynomial and RBF kernels. All classification accuracies were averaged across the twelve participants.

Kernel type	Linear	Polynomial	RBF
All movement conditions (type and frequency)	56.6 ± 9	81.3 ± 0.7	79.5 ± 4

#### Validation

Through a one-sided Wilcoxon test, the results of both comparing the accuracies of texture classification problem with (a. movement type, and b. movement frequency) showed the rejection of the null hypothesis (equal accuracies) with a statistical significance p-value = 2.0568e−05. The effect size of (1. texture classification and 2. movement Type classification accuracies) is 0.9. While, The effect size of (1. Texture classification and 2. movement frequency classification accuracies) is 2.1.

The result of the one-sided Wilcoxon test applied in the second step of validation was rejecting the null hypothesis with a statistical p-value = 1.8 e−05. The decision boundaries selected by the SVM classifier for each classification problem and the separability of each class per condition are shown in Fig. S1 in the supplementary material.

## Discussion

The research objective of this study is to identify and analyze trial-based salient EEG features associated with active touch of surfaces having varying degrees of roughness. For this purpose, the study protocol involved tactile stimuli of synthetic textured surfaces with different levels of roughness (flat, medium-rough and rough surfaces). The active touch exploration was performed using three levels of movement frequency (approximately 2, 1 and 0.5 Hz) and two types of movement conditions (rub and tap).

During the systematic feature selection process, a subset of spectral EEG features was selected, see Fig. 1 and the performance of the feature subset marked with a red arrow. These features reflect the changes in the cortical activity during active exploration of tactile stimuli. During active touch, the elicited EEG responses are influenced by both the motor and sensory aspects of the task. The motor component’s contribution to the EEG response may affect the accuracy of the texture identification. Therefore, in order to minimize the influence of both movement type and frequency level on the texture identification accuracy, our approach includes a systematic feature selection that is sensitive to variations in texture while including minimal to no influence from movement conditions (type and frequency). Therefore, a systematic approach is taken to select features that contribute to texture classification, and at the same time, minimize the accuracy of classifying different movement type and frequency conditions through EEG.

An initial set of temporal and spectral features were selected based on previous studies of cortical activity related to the exploration of tactile stimuli. Then, a subset of these features were chosen based on their ability to provide sensory information about different textures^18,19,24^. More specifically, we considered the normalized total power and the average power in the theta (3–6 Hz), mu (7–12 Hz), beta (13–30 Hz) and gamma (> 0 Hz) bands, the average EEG amplitude and P300 response of each trial as the EEG features and applied a forward sequential feature selection such that our approach started with a single feature and added other features satisfying high texture identification accuracy and low movement type and frequency classification accuracy to the list of features. The identified set includes total power in the beta and mu frequency bands, and results corresponding to this set of features are presented in Figs.  2, 3 and  4, specifically 84 ± 2.4% (chance level 38.3%), 60 ± 2.4% (chance level 45.3%) and 57 ± 3.2% (chance level 39.9%) mean texture, movement type and frequency classification accuracies are obtained.

The results of Fig. 2 show that the highest texture classification accuracy values are obtained around the somatosensory region of the brain contralateral to the stimulated fingertip. These results align with the findings reported in previous studies^17,22^. Moreover, for both the rub and tap conditions, the sensitivity of discriminating a textured surface increased with the increase in roughness level. This reflects the importance of the roughness in the perception and discrimination of textured surfaces as has been shown previously^25,26^. Additionally, the cortical processing of roughness discrimination follows two schemes, cognitive and sensory-based processing. The first generates activation in the prefrontal areas while the second involves mostly the somatosensory region^10^. As can be observed in Fig. 2B, fast touching conditions activate prefrontal areas in addition to the somatosensory regions that are contralateral to the stimulated fingertip. The overall average movement condition classification accuracies across all participants for all the conditions range between 60 and 65 % (chance level is 45.3%). The sensitivity of the rub movement is higher than the tap movements for all touch conditions (Fig. 3A). Thus, by increasing the time spent contacting the surface, the cortical response to the tactile stimulus provided greater discriminability for the presented textures. Similarly in the tap condition, the slow movement frequency achieved higher accuracy than both fast and medium movement frequencies. This could reflect an increase in the EEG data’s ability to classify textures as touching movement frequency decreases. The scalp topography maps show a slight increase in the average accuracies over the sensorimotor regions. This could be due to the motor component in the selected set of features. Therefore, these features could discriminate movement conditions but with low accuracy values. The performance of the movement frequency classification showed overall average accuracy between 45 and 58% (chance level is 39.9%). In both movement conditions, the movement frequency discrimination accuracy increased with the increase in the textured surface roughness level (Fig. 4A).

In order to validate the efficiency of the chosen set of features in discriminating textured surfaces with minimal influence from the movement type and frequency conditions, three classification problems have been formed, and the results are presented in Table  1. The first two were performed by combining the EEG trials of all the movement frequency conditions per movement condition. That is, three classes of EEG trials are formed: (class 1) tap smooth flat surface with low, medium and fast movement frequency; (class 2) tap medium rough surface with low, medium and fast movement frequency; and (class 3) tap rough surface with low, medium and fast movement frequency). Similarly, for the rub movement, three classes have been formed and used in the classification. For the third classification problem, we combined all the EEG trials belonging to the same texture regardless of the movement type and frequency conditions forming the following three classes: (class 1) rub or tap flat surface with low, medium and fast movement frequency; (class 2) rub or tap medium rough surface with low, medium and fast movement frequency, and (class 3) rub or tap rough surface with low, medium and fast movement frequency. The overall average accuracy across all participants showed that despite combining the movement type and frequency conditions, the chosen set of features were able to discriminate between the textured surfaces with average accuracy greater than 81% and low values of sensitivity, Table 1. Moreover, the accuracy of texture classification increased with the rub movement and as the roughness of the surface increased.

## Materials and methods

### Experimental procedure

#### Participants

Twelve right-handed healthy participants (4 males, $26 \pm 3.4$ years) with no neurological or somatosensory deficits, physical limitation or skin rash were recruited in this study. The study was approved by University of Pittsburgh ethics committee/IRB (IRB # STUDY19020352). Written Informed consent was provided by all the participants involved in the study. During the consent process the participants were informed that their safety and the confidentiality of the collected data are the primary consideration. Participants were told that at any point during the experimental procedure, if they feel any discomfort, they could stop the experiments. Moreover, all the experimental procedures described below follow directly the approved IRB. All methods were carried out in accordance with relevant guidelines and regulations.

#### Tactile stimuli

**Figure 5:**
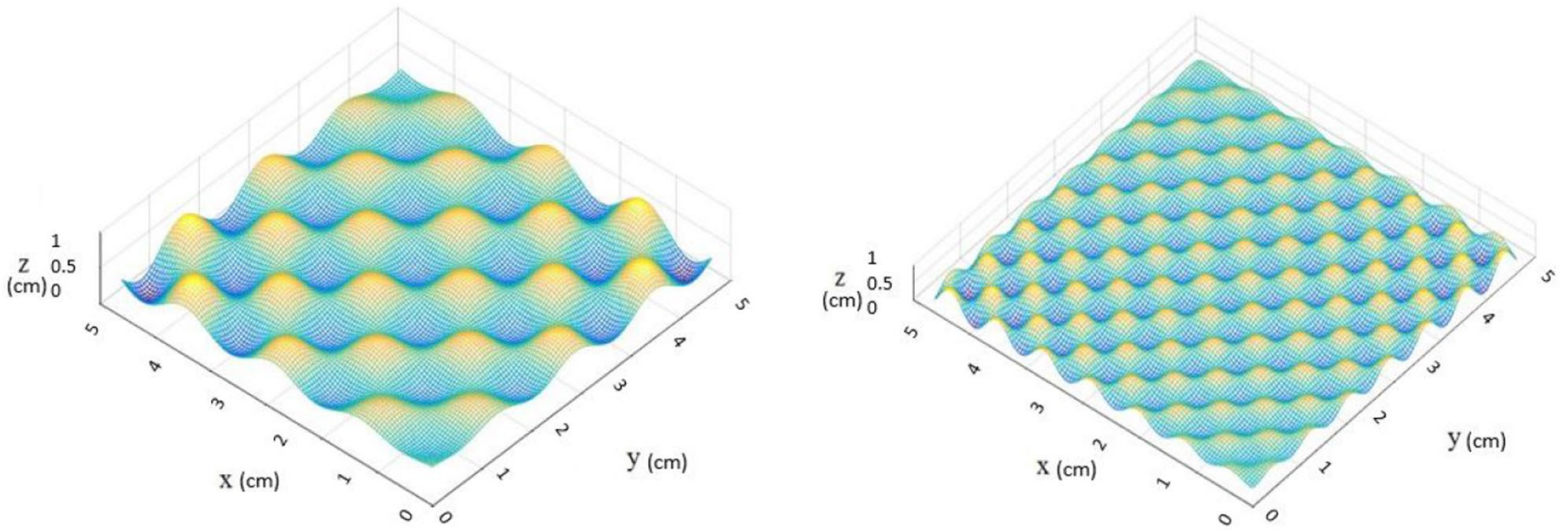
The power spectral density of the medium rough and rough surfaces respectively.

The tactile stimuli used in this study represent three levels of roughness. A set of three textures (5 cm $\times$ 5 cm) have been generated using MATLAB and fabricated with Stereolithography (Viper SLA system, 3Dsystems, USA). Each one of the three surfaces represents a different level of roughness, ranging from a smooth flat surface to a rough surface. The power spectral density which controls the level of roughness of each surface is given by :

1 $$\begin{aligned} \phi (|k|) = {\left\{ \begin{array}{ll} C, &{} \text {if}\,k_l<= |k|<= k_r.\\ C \left( \frac{|k|}{k_r}\right) ^{-2(1+H)}, &{} \text {if}\, k_r<= |k| <= k_s.\\ 0, &{} \text {otherwise}. \end{array}\right. } \end{aligned}$$

where *C* is the roughness amplitude, $k_l$, $k_r$, $k_s$ are the lower roll-off and upper cutoff wave numbers and *H* is the Hurst roughness exponent. Figure 5 shows both the medium rough (H = 0.5, $\hbox {C} = 10 \times 10^{10}$ $k_l = k_r = 16$, $k_s = 64$) and rough (H = 0.5, $\hbox {C} = 10 \times 10^{10}$ $k_l = k_r = 32$, $k_s = 256$) surfaces used in this study. Each texture is mounted on a force transducer and adjusted on a table, and the participants were comfortably seated in front of the system setup.

#### Data acquisition

EEG was recorded according to the 10–20 system from 14-channels, using electrodes placed over the frontal and somatosensory cortices focusing around the sensorimotor integration regions (F3, F4, FC3, FC4, C1, C3, C5, CZ, C2, C4, C6, CP1, CPZ and CP2). The left mastoid was used as a reference and FPz as the ground electrode. In this study, two g.USBamp (from g.tec medical engineering GmbH) amplifiers were used, one for EEG data acquisition and one for force data. Recorded EEG data were digitized with 1200 Hz sampling rate. EEG signals were filtered using a 4th-order notch filter with cut-off frequencies of 58 and 62 Hz, and an 8th-order bandpass filter with cut-off frequencies of 2 and 62 Hz. EEG data was further preprocessed using FIR bandpass filter designed using Kaiser window with cut-off frequencies of 8 and 60 Hz. A force and torque transducer (NANO17 F/T transducer, ATI Industrial Automation, USA) was used to record force data. The force data was then transferred to the analog inputs of the g.USBamp amplifier and sampled at 1200 Hz. The two amplifiers were connected to each other to enable synchronization across EEG and force data. Moreover, both EEG and force data were synchronized to each condition through a digital trigger. Cues were presented to the participant using Psychtoolbox (MATLAB)^27^ and an event marker was sent to each amplifier to mark the time of cue onset. The event markers were then used to segment both the EEG and force data per condition. In this experiment, we have a total of 18 conditions: a combination of texture surface, movement frequency and type. Within a condition, the participant was instructed to rub or tap the chosen surface multiple times with a specific movement frequency. Each complete rub or tap movement was considered a single trial. Trials were segmented using the normal contact force component. After the cue and during the touch condition, a black screen was shown. After the completion of a condition, a “Rest” message was presented for one minute.

**Figure 6:**
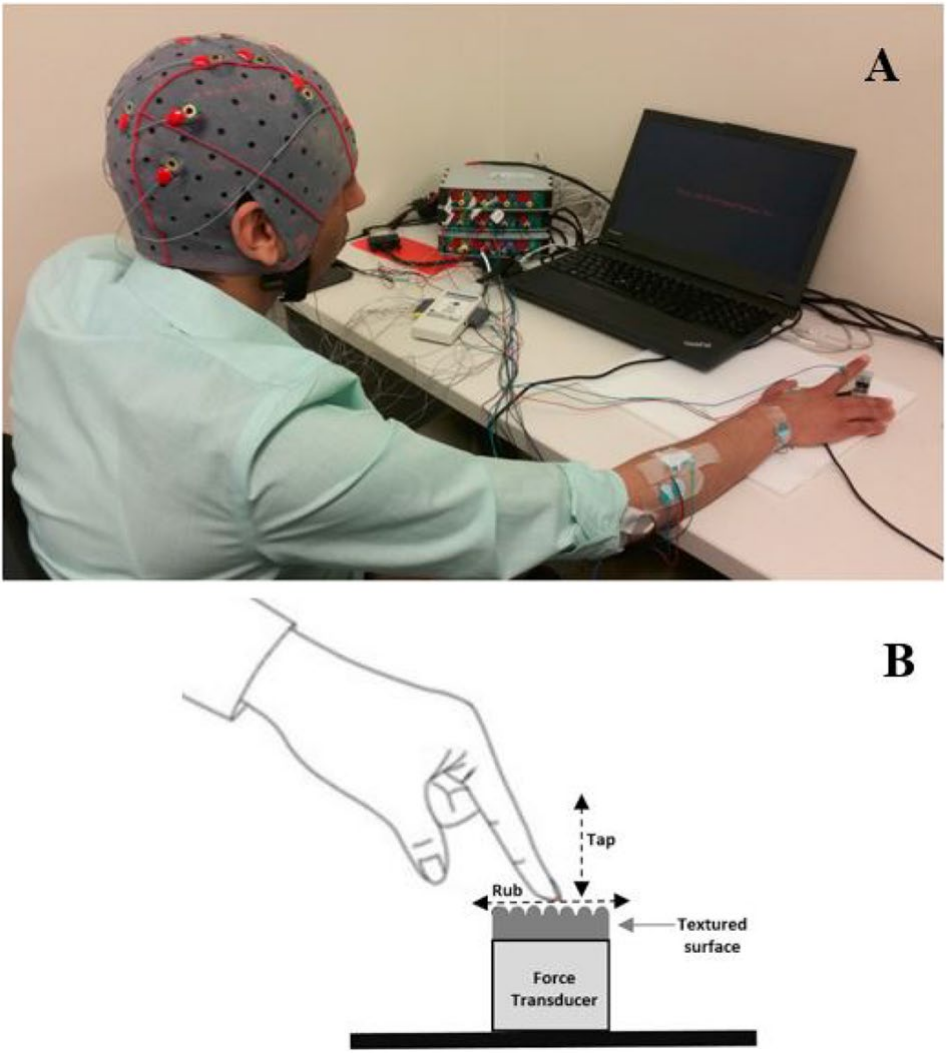
Experimental Setup. (**A**) The participant is tapping the texture mounted on the force transducer. (**B**) Schematic diagram showing the tap and rub movement conditions.

#### Experimental setup

**Figure 7:**
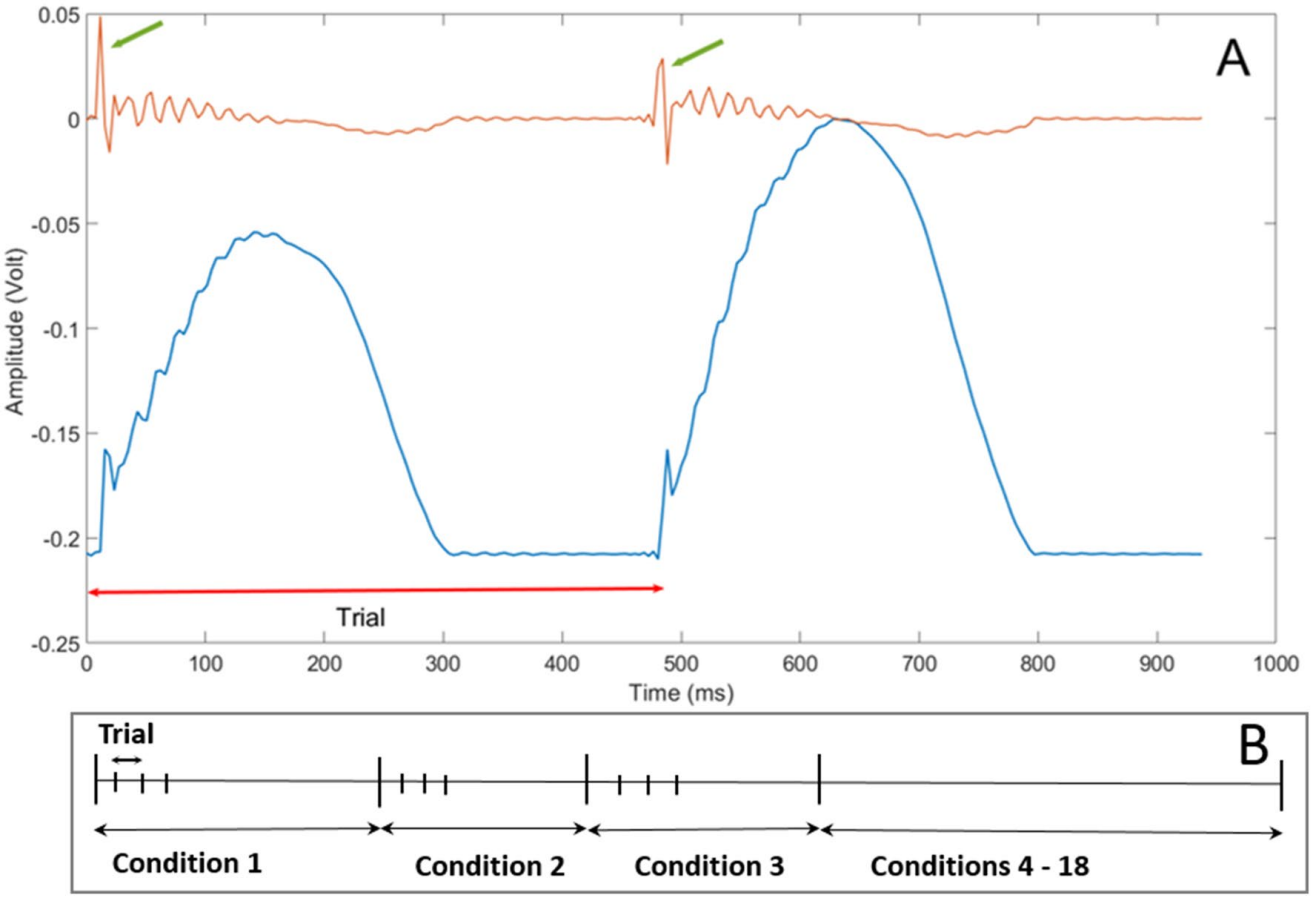
(**A**) The normal force profile, in blue, and the result of applying a first order derivative filter in red. The green arrows show the local peaks that mark the beginning and end of each trial. (**B**) An illustration showing the 18 conditions, and the trials within each condition.

The experimental setup is shown in Fig. 6. During the experiment protocol, the subjects were asked to sit comfortably in front of the system setup and rest their right arm on the table as shown in Fig. 6. In order to avoid any visual or auditory distractions, the participants were asked to look at a black screen presented on the computer in a quiet room. Three surfaces with different levels of roughness (smooth flat, medium rough and rough surfaces) were used. These surfaces were securely attached to a force transducer which was fixed on a table. The measured contact forces and EEG were recorded synchronously while the participant was rubbing or tapping each surface for one minute. We have 18 different conditions, a combination of movement type (rub/tap), movement frequency (slow, medium frequency, and fast) and three textures (smooth flat, medium rough, and rough). Each participant was instructed to rub or tap each surface with one of three different movement frequencies (0.5, 1, and 2 Hz) for one minute. The 18 conditions were randomized for each participant, and there was one minute of rest after each condition. Trigger values are used to segment both the EEG and force data per condition. We denoted one complete rub/tap over the surface as a trial and used the recorded normal contact force component to segment the EEG for different trials, as can be seen in Fig. 7.

### Data analysis

#### Force data analysis

Careful examination of the normal contact force component, Fz, showed significant pattern to each fingertip movement. This normal force component was analyzed to segment each trial, which denotes a complete rub or tap movement. For each condition, a series of preprocessing steps have been applied. First, a first order derivative filter was applied followed by a local maxima detection algorithm. The resulting local peaks mark the beginning and end of trials, as shown in Fig. 7A. Finally, careful observation of the force segmented data was carried out to ensure the correctness of segmentation. The segmented force data was then used to segment the corresponding EEG trial data and to calculate the average movement frequency per touch condition.

**Table 3:** The mean and standard deviation of the average movement frequency, trial length and average peak force amplitude of all the 12 participants during each touch condition.

Movement	Movement	Surface	Movement	Trial length	Force amplitude	Number of trials
Type	Frequency	Type	Frequency (Hz)	(ms)		
Rub	Fast	Flat	1.95 ± 0.17	484 ± 88	0.047 ± 0.014	117 ± 10
Rub	Fast	Medium rough	2.00 ± 0.22	464 ± 93	0.038 ± 0.014	120 ± 13
Rub	Fast	Rough	1.83 ± 0.19	588 ± 82	0.034 ± 0.017	110 ± 11
Rub	Medium	Flat	1.17 ± 0.11	873 ± 88	0.052 ± 0.023	70 ± 7
Rub	Medium	Medium rough	0.98 ± 0.14	1049 ± 86	0.045 ± 0.019	58 ± 8
Rub	Medium	Rough	0.97 ± 0.1	908± 120	0.025 ±0.013	58 ± 6
Rub	Slow	Flat	0.6 ± 0.18	1843 ± 120	0.026 ±0.015	36± 10
Rub	Slow	Medium rough	0.62 ± 0.17	1884 ± 130	0.048 ±0.024	37 ± 5
Rub	Slow	Rough	0.58 ± 0.08	1865 ± 109	0.036 ±0.018	35 ± 10
Tap	Fast	Flat	2.17 ± 0.17	464 ± 56	0.097 ± 0.015	130 ± 10
Tap	Fast	Medium rough	2 ± 0.18	409 ± 80	0.098 ± 0.020	120 ± 11
Tap	Fast	Rough	1.92 ± 0.165	492 ± 69	0.11 ± 0.020	115 ± 10
Tap	Medium	Flat	1.17 ± 0.05	1177 ± 120	0.09 ±0.014	70 ± 3
Tap	Medium	Medium rough	1.04 ± 0.045	872 ± 120	0.1 ± 0.020	62 ± 3
Tap	Medium	Rough	1.08 ± 0.06	834 ± 103	0.11 ± 0.018	65 ± 4
Tap	Slow	Flat	0.62 ± 0.06	1839 ± 208	0.09 ± 0.03	38 ± 4
Tap	Slow	Medium rough	0.63 ± 0.056	1873 ± 127	0.128 ± 0.019	37 ± 3
Tap	Slow	Rough	0.50 ± 0.05	1735 ± 113	0.110 ± 0.02	30 ± 3

#### EEG data analysis and feature extraction

The indices of the segmented force data were used to mark the 14 channels of the recorded EEG. For each trial, we extracted features based on the temporal and spectral properties of the corresponding EEG segment^24,28^. More specifically, the normalized total power and in the theta (3–6 Hz), mu (7–12 Hz), beta (13–30 Hz) and gamma (>30) bands were calculated. Welch periodogram method of power spectral estimation was used to calculate frequency-based features^29,30^. Also, the average EEG amplitude and P300 response, which is a positive change in the EEG around occipital-parietal recording sites around 300 ms after the stimuli, of each trial were calculated^31^. These features were standardized and concatenated to form a feature vector that was used in classification. We also removed the outliers from each participant’s dataset. We define an outlier as an element that has a value more than three standard deviations from the mean. The process of outlier rejection resulted in rejecting $4 \pm 2 \%$ of the data trials.

#### Movement frequency distribution

In order to have a uniform movement frequency distribution across all the participants per each movement frequency level, a training session was carried out before each experiment. During this session, the participant learned to rub or tap the three textured surfaces at three movement frequency levels (2, 1 and 0.5 Hz). After finishing the training session, the experimenter did not intervene to instruct the participant to modify his movement frequency during the experiment. The normal component (z-axis) of force data collected during each touch condition was segmented per trial. This segmented data was then used to estimate the average movement frequency per condition. The mean and standard deviation of the average movement frequency of all 12 participants during each touch condition is shown in Table  3. The movement frequency is uniformly distributed around the three levels (2, 1 and 0.5 Hz) with low variance.

#### Classification

A Support Vector Machine classifier (SVM) was used to evaluate the effectiveness of the selected features in discriminating surface texture^32^. SVM basically aims to find an optimal hyper-plane (usually through kernel transformation to enable linear separation) that categorizes new observations by formulating an optimization problem using the training observations. In this study, a second order polynomial kernel was used. A 3-class SVM classifier was used to discriminate between the textured surfaces (flat, medium rough, and rough). Another 3-class SVM classifier was used to discriminate between the movement frequency conditions. Finally, we trained a 2-class SVM classifier to classify between the movement conditions (rub and tap). We used tenfold cross-validation to train the SVM classifiers. In order to calculate the chance level of each classification problem, we randomly permuted the labels and reversed each trial and shift it randomly within the range (0–300 ms). Then we calculated the average accuracy for each classifier for the twelve participants. The results showed that the chance levels are as follows : Texture classification $38.3\% \pm 3.8$, Movement type $45.3\% \pm 4.3$ and movement frequency $39.9\% \pm 4.5$. Furthermore, a comparison between the performance of the (linear, polynomial and RBF kernels) for the texture classification problem has been also performed, and the results are shown in Table  2.

A systematic method along with the sequential feature selection method were used to select the features that contribute to texture classification but have low contribution towards movement frequency and type classification. This method has two main components, an objective function and a sequential forward search algorithm. For the objective function we used the misclassification rate to minimize over all feasible feature subsets for texture classification. The search algorithm has been chosen in a way that increases the feature candidate set which maximizes the total texture identification classifier accuracy. It also minimizes the total movement type and frequency classifier accuracies. A detailed analysis of each feature’s contribution towards the classification of texture, movement type and frequency has been done. The selected six features were used for this analysis, (the total power in the theta band, the total power in the mu band, the total power in the beta band, the total power in the gamma band, the average EEG amplitude and the P300 response). Six different groups of feature subsets have been generated, groups 1 to 6. Each group can be described as follows, the first group contains six subsets of features, each subset consists of one feature from the six previously mentioned features. The second group consists of 15 combinations of two pairs of features, so for example a subset in this group consists of the following two features (the total power in the theta band and the total power in the beta band). Similarly, the third group consists of 20 combinations of three features, so a subset of this group would look like this (the total power in the theta band, the total power in the mu band and the total power in the beta band). Group four, consists of 15 combinations of four features, while group five consists of 6 combinations of five features. Finally, group six contains all the six features under study. Each subset of features, total of 63 subsets of features, is used in the three classification problems (texture, movement type and frequency).

#### Validation

In order to assess the results of the classification problems, we performed one-sided Wilcoxon rank statistical test between the accuracies of the texture classification and both the movement type and frequency. The Wilcoxon rank test^33^ is a nonparametric hypothesis test, which returns the p-value for the null hypothesis. The null hypothesis states that the median of the accuracies of the texture classification problem is not greater than that of (a. movement type, b. movement frequency). While, the alternative hypothesis states that the median of the accuracies of the texture classification problem is greater than that of (a. movement type, b. movement frequency). To evaluate the significance of the accuracies of classifying the textures versus the accuracies of both movement type and frequency conditions. The accuracy vector containing the accuracies of each classifier obtained from the 12 participants were used.

We also calculated Cohen’s d^34^ which represents the effect size for the Wilcoxon rank test. Specifically, we calculated the following:

2 $$\begin{aligned} d= \left( Median_{Group1} - Median_{Group2}\right) / SD_{pooled} \end{aligned}$$

And the standard deviation is calculated as follows:

3 $$\begin{aligned} SD_{pooled} = \sqrt{ \left( \left( SD_{group1}\right) ^{2} + \left( SD_{group2}\right) ^{2} \right) /2} \end{aligned}$$

A second step of validation was carried out by randomly permuting the labels and reversing each trial and shift it randomly within the range (0–300 ms). Then calculating the accuracy of textured surfaces classification for all movement types and frequencies. We used one-sided Wilcoxon rank statistical test between the accuracies of the texture classification between the original trials and the same set of trials permuted and shifted with random amount of time. The null hypothesis in this case states that the median of the accuracies of the texture classification problem of the (1) original non permuted and non shifted set of trials is not greater than that of (2) the same trial set with random permutation and time-shifting. While, the alternative hypothesis states that the median of the accuracies of the texture classification problem of the first set of trials is greater than that of the second set of trials. The accuracy vector containing the accuracies obtained from the 12 participants were used for both sets of trials.

### Ethical statement

All methods were carried out in accordance with relevant guidelines and regulations.

## Conclusion

The future goal of this work is to develop a system which could mimic the sensation of various textured surfaces guided by EEG during different applications such as: surgical training of physicians in virtual environment, remote control of robotic arms and teleoperations. Therefore, we investigate in this study the possibility of extracting trial-by-trial EEG features that could classify textures independent from movement type and frequency conditions.

In this paper, we showed that using EEG it is possible to classify different textures during active touch. Results showed that EEG features based on the total powers in mu and beta frequency bands enabled classification of textures independent from movement type and frequency conditions with very high accuracy on a single trial basis. The accuracy increased with lower movement frequency of interaction and as the roughness of the surface increased.

In summary, the aim of this study was to develop analysis techniques to extract EEG features that could classify various textures with different levels of roughness using EEG. As part of the future work, we aim to develop an automated feature extraction and selection which could extract sensory related information while suppressing the influence of movement conditions. For this future direction we will investigate two different approaches: control systems through state space modeling^35–38^ and generative adversarial networks^39^. The selected set of EEG features will be used to design principles for model-based optimal EEG-guided closed-loop haptic feedback system in our future work.

## Supplementary information

Supplementary Information.
