# Supplementary material for: EEG-based trial-by-trial texture classification during active touch
Source: Sci Rep. 2020 Nov 27;10:20755. doi: 10.1038/s41598-020-77439-7 (PMC7699648; doi:10.1038/s41598-020-77439-7)
Supplement: Supplementary file 1 — Supplementary Information. [file 41598_2020_77439_MOESM1_ESM.pdf]

EEG-based Trial-by-Trial Texture Classification during Active Touch

Safaa Eldeeb[1*], Douglas Weber[2], Jordyn Ting[2], Andac Demir[3], Deniz Erdogmus[3], and Murat Akcakaya[1]

[1]Swanson school of Engineering, Electrical and Computer Engineering Department, University of Pittsburgh, PA, USA.

[2]Swanson school of Engineering, Bioengineering Department, University of Pittsburgh, PA, USA.

[3]Electrical and Computer Engineering Department, Northeastern University, MA, USA.

*sme46@pitt.edu

The selected set of EEG features (the total power in the Mu (8-15 Hz) and Beta (16-30 Hz) frequency bands) showed contribution towards the texture classification (sensory information) and low contribution towards movements conditions, type and frequency, (motor part). In the following figure we show the changes in both EEG features and how they are perceived by each classification problem. We start with each classification problem conditioned on specific conditions, as shown in supplementary Fig. S1. Specifically, supplementary Fig. S1.A shows one example of the 3-class texture classification problem for a specific movement condition (rub and fast), while supplementary Fig. S1.B shows 2-class movement type condition classification (rub vs tap) and supplementary Fig. S1.C shows 3-class movement frequency condition classification (fast vs medium vs slow). In supplementary Fig. S1.A, we show the different texture classes conditioned (rub and fast movement conditions) for one of the participant's datasets. The x-axis represents the power in the Mu EEG frequency band, while the y-axis represents the power in the Beta band. Each point represented by a circle represents the Power in Mu and Beta at each single trial. We applied SVM classifier, and drew the decision boundaries for each problem. As we can see in supplementary Fig. S1.A, the three classes, flat (blue), medium rough (red) and rough (green) surfaces, are separable with low number of outliers for each class. The light grey, grey and dark grey regions represent the flat, medium rough and rough surfaces classes regions, respectively, generated using the SVM classifier. We can see that these regions and their decision boundaries were able to separate the three classes successfully with minimal error. While on the other hand, supplementary Fig. S1.B shows the inseparability of the two classes (rub vs tap) for the same set of features (power in Mu and Beta). Moreover, Fig. S1.C shows the inseparability of the three classes (fast, medium and slow) for the same set of features. These figures S1. (A-C) show that the selected set of features are able to successfully classify textures with minimal influence of the movement conditions. As we mentioned, our goal is to maximize texture classification while minimizing movement conditions classification accuracy, which can be shown in this figure by minimizing the misclassification rate of the texture classification problem (represented by the separability of the three classes) and maximizing the other two movement conditions misclassification rate (represented by the significant overlap and minimal separability of the movement classes).

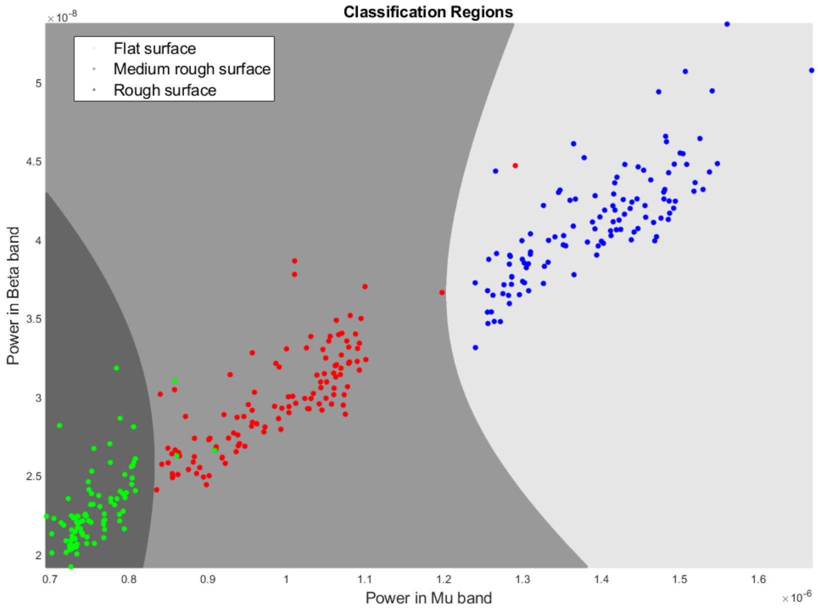

Supplementary figure S1.A The decision boundaries selected by the SVM classifier for texture surfaces classification problem. Blue colored circles represent flat smooth surface, red colored circles represent medium rough surface and green colored circles represent rough surface. The light grey, grey and dark grey regions represent the flat, medium rough and rough surfaces classes regions, respectively, generated using the SVM classifier.

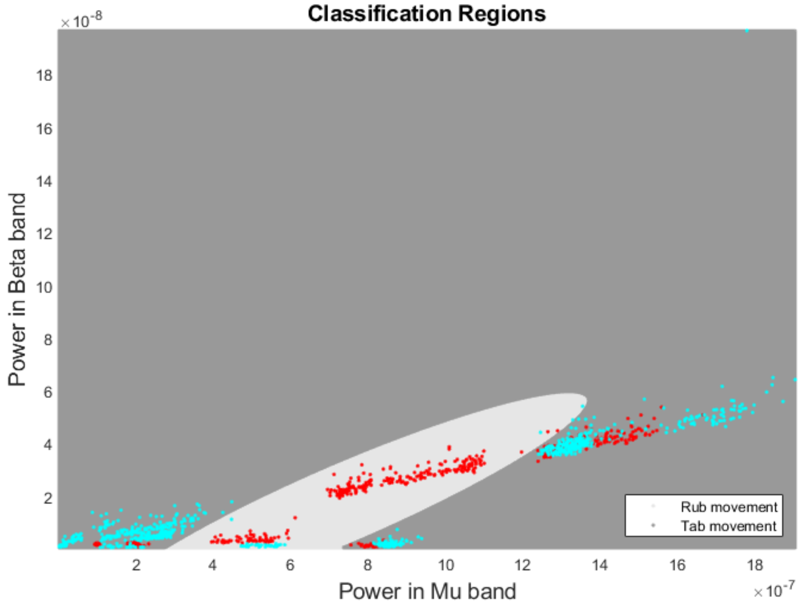

Supplementary figure S1.B. The decision boundaries selected by the SVM classifier for movement type conditions classification problem. Light blue colored circles represent tap movement and red colored circles represent rub movement condition. The light grey and dark grey regions represent the tab and rub movement classes regions, respectively, generated using the SVM classifier.

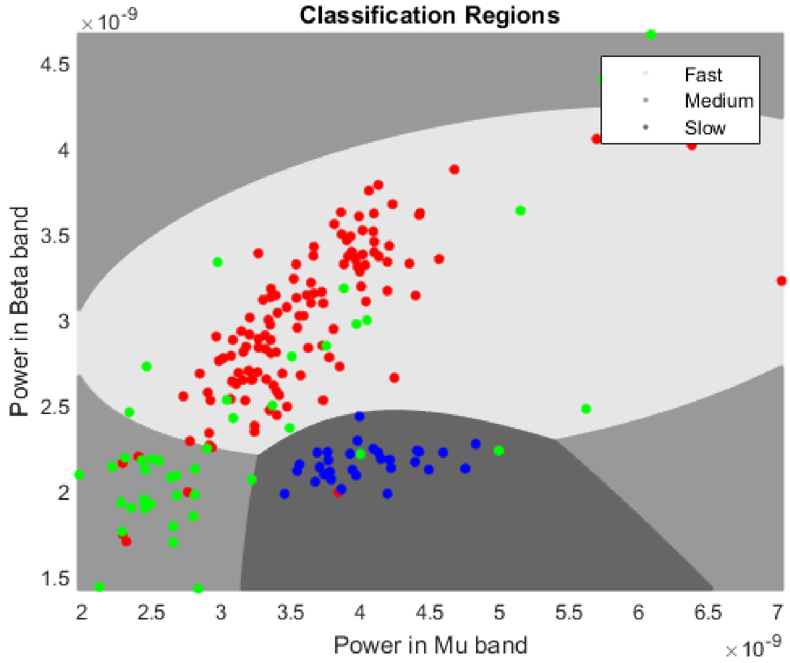

Supplementary figure S1.C. The decision boundaries selected by the SVM classifier for movement frequency conditions classification problem. Red colored circles represent fast movement frequency, green colored circles represent medium frequency and blue colored circles represent slow frequency. The light grey, grey and dark grey regions represent the fast, medium and fast movement frequency classes regions, respectively, generated using the SVM classifier.
